# Neural circuitry involved in quitting after repeated failures: role of the cingulate and temporal parietal junction

**DOI:** 10.1038/srep24713

**Published:** 2016-04-21

**Authors:** Weihua Zhao, Keith M Kendrick, Fei Chen, Hong Li, Tingyong Feng

**Affiliations:** 1 School of Psychology, Southwest University, Chongqing 400715, China; 2 Key Laboratory for Neuroinformation, Center for Information in Medicine, School of Life Science and Technology, University of Electronic Science and Technology of China, Chengdu 610054, China; 3 School of Psychology and Sociology, Shenzhen University, Shenzhen 518060, China; 4 Key Laboratory of Cognition and Personality, Ministry of Education, Chongqing 400715, China

## Abstract

The more times people fail the more likely they are to give up, however little is known about the neural mechanisms underlying this impact of repeated failure on decision making. Here we have used a visual shape discrimination task with computer-controlled feedback combined with functional magnetic resonance imaging (fMRI) to investigate the neural circuits involved. The behavioral task confirmed that the more times subjects experienced failure the more likely they were to give up, with three successive failures being the key threshold and the majority of subjects reaching the point where they decided to quit and try a new stimulus set after three or four failures. The fMRI analysis revealed activity changes in frontal, parietal, temporal, limbic and striatal regions, especially anterior cingulate cortex (ACC), posterior cingulate cortex (PCC) and temporal parietal junction (TPJ) associated with the number of previous failures experienced. Furthermore, their parameter estimates were predictive of subjects’ quitting rate. Thus, subjects reach the point where they decide to quit after three/four failures and this is associated with differential changes in brain regions involved in error monitoring and reward which regulate both failure detection and changes in decision-making strategy.

Reinforcement learning (RL) is a process whereby the estimation of state/action values is improved through trial and error so that behavior becomes more advantageous^1^. The detection and evaluation of behavioral feedback are thus of critical importance to allow adjustment of subsequent choices efficiently^2^,^3^,^4^,^5^,^6^. For example, failure (negative feedback) is common and can be used to shift from one set of stimulus-response translation rules to another^7^. In many situations optimal behavior requires the selection of actions based on the expected value that is derived from an individual’s recent history of failures^8^,^9^. Thus, feedback on failure is important for making subsequent choices and plays a key role in influencing behavioral decision making. For example, learned helplessness is suggested to occur when an individual’s desire to learn is destroyed because of unpleasant and uncontrollable events.

To behave adaptively, animals and humans need the capacity for reinforcement learning to internally monitor responses and to evaluate external reinforcement or feedback^10^, and learn by trial and error to act in a manner that maximizes reward and minimizes punishment^11^,^12^. This process applies to many different situations and appears to be supported by subcortical and cortical neural networks^13^,^14^,^15^,^16^,^17^. Widespread neural systems are involved in encoding the value of outcomes received and responses related to monitoring or evaluation of a previously executed action. The main regions implicated include the anterior cingulate cortex (ACC), medial prefrontal cortex (MPFC), dorsolateral prefrontal cortex (DLPFC), amygdala, ventral striatum (VStr), posterior cingulate cortex (PCC) and temporal parietal junction (TPJ)^18^,^19^,^20^,^21^. However, previous studies have focused on the effects of a single feedback given on a trial by trial basis^4^,^21^,^22^,^23^ rather than on how decision making, and associated neural mechanisms, are impacted by the more common experience of repeated failure during the performance of a task.

The “three strikes” phenomenon is well known and has often been reported in research related to decision making or sports psychology^24^,^25^. This phenomenon relates to the so called “hot hand” belief in sport where a player is considered to have a higher chance of making a shot after two or three successful shots than after two or three misses (resulting in “streaks”). However, in all these previous papers it is clear that the paradigms used resulted in subjects having an increased expectation of success after each failure. The current study aimed to identify more precisely what cognitive rules operate and which neural networks are involved in monitoring performance and predicting choice in order to regulate decision making in the face of repeated failures, and where each failure is not associated with a greater expectation of subsequent success. We used a novel feedback visual discrimination paradigm^26^ with computer-controlled feedback, where the subject could either decide to continue (try again) or abort (quit and try a new discrimination) after failing on a trial. In the study we focused on the critical number of sequential trials on which subjects failed before deciding to quit and try a new stimulus set. Importantly, the paradigm deliberately minimized the potential for modulatory influences of situations where subjects could either achieve high gains (such as in gambling situations) or had other strong motivations (such as providing solutions to problems of significant intrinsic interest) for persisting with solving a task which might have resulted in an increased and highly variable tolerance of repeated failure. We hypothesized that subjects would reach a threshold after a specific number of failures when they would be more likely to change from persisting with the current stimulus set to giving up and trying a new stimulus set. We further hypothesized that the frontal and limbic brain regions specifically involved in decision making and controlling the effects of repeated failure would be involved, and exhibit changes in activity and functional connectivity associated with behavioral performance.

## Results

### Relationship between negative feedback and quitting rate

Trial-by-trial analysis revealed that whether subjects decided to continue or quit discrimination of a specific stimulus set was influenced by the number of times they received negative feedback. We used a logistic regression model to predict whether subjects continued or gave up after receiving different numbers of negative feedbacks. This simplified model used one factor (the number of negative feedbacks) and could be expressed as


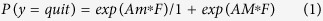


with the following functions: Logit(P) = Am*F, where F is the number of negative feedbacks, Am is the regression coefficient of negative feedback. The indices of the model were: Cox & Snell *R*^2^ = 0.286, Nagelkerke *R*^2^ = 0.451, accuracy of prediction = 84.2%. Based on the model, we drew a ROC curve with an area under the curve of 0.8 < S = 0.869 < 0.9, suggesting that the model diagnosis was good (*S.E.* = 0.008, *p* < 0.001, 95% confidence interval is 0.854–0.884). Using Hosmer-Lemeshow analysis to test goodness of fit we found that there was no difference between the prediction result and initial data, which also indicated that the model was good (*χ*^2^ < 0.001, *df* = 3, *p* > 0.05). These results showed that whether to continue or to quit was indeed influenced by the number of times subjects received negative feedback.

**Figure 1:**
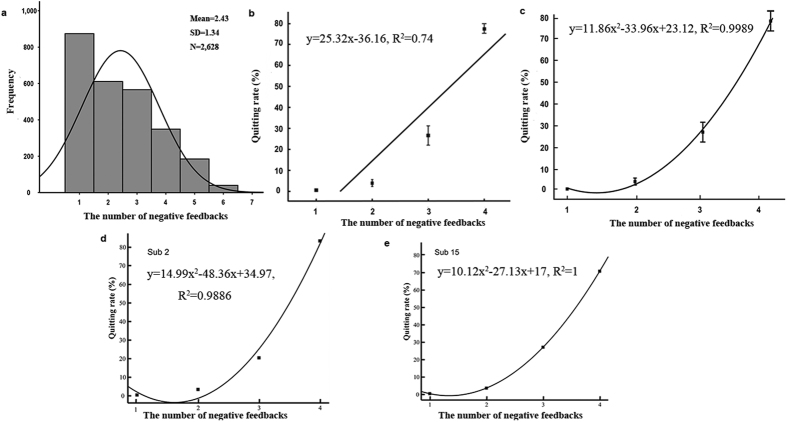
Behavioral results. (**a**) The coverage of choosing to continue. (**b**,**c**) A linear regression (**b**) and a quadratic polynomial (**c**) function between the number of negative feedbacks and quitting rate. (**d**,**e**) The relationship between the number of negative feedbacks and quitting rate using a quadratic polynomial function in two representative subjects.

Descriptive statistics showed that for the largest proportion of trials (91.33%) the decision to quit occurred between 1 and 4 failures, with most subjects doing so after 3 or 4 failures. Only 7.04% of quitting decisions occurred after 5 failures, 1.52% after 6 and 0.11% after 7 (see Fig. 1a). In the subsequent analyses we therefore combined trials where a 4^th^, 5^th^, 6^th^ or 7^th^ negative feedback was received. With respect to quitting rate, a repeated-measures ANOVA revealed a main effect of the number of negative feedbacks, *F*_(*3*,*57*)_ = 206, *p* < 0.001. Post-hoc multiple Bonferroni-corrected comparison analyses indicated that there were significant differences between the each of the different numbers of negative feedbacks received (*M1* = 0.57%, *SD1* = 1.26%, *M2* = 3.98%, *SD2* = 7.2%, *M3* = 26.61%, *SD3* = 20.48%, *M4* = 77.44%, *SD4* = 9.62%, all *p* < 0.001), other than between the first and second time (*p* = 0.170). These results showed that subjects reached the point where they started to change from continuing to quitting after the third time of receiving negative feedback. To further explore the relationship between the number of times negative feedback was received and quitting rate (percentage of quitting the item), we conducted a linear regression (*y* = *ax* + *b*) and a quadratic polynomial curve (*y* = *ax*^2^ + *bx* + *c*) separately. The linear regression function was *y* = 25.32*x* − 36.16, *R*^2^ = 0.74 (see Fig. 1b). The fit of the quadratic polynomial function was also good: *y* = 11.86*x*^2^ − 33.96*x* + 23.12, *R*^2^ = 0.9989, 95% confidence intervals (see Fig. 1c). To illustrate this finding, Fig. 1d,e show the quadratic polynomial function for quitting rate in individual trials as a function of the number of negative feedbacks in two representative subjects. Since the quadratic polynomial function was superior to the linear regression one we used it in all subsequent analyses. Overall, these results suggested that quitting rate was predicted by the number of negative feedbacks received and reached the point where subjects started to decide to quit rather than continue after a third failure with most quitting after 4 or more failures.

An analysis of confidence levels after each failure showed that they decreased following each successive failure (see supplementary information and Fig. S1). Importantly this confirmed that in our paradigm subjects did not experience an increased expectation of subsequent success after each failure.

### Neural correlates of negative feedback

**Figure 2:**
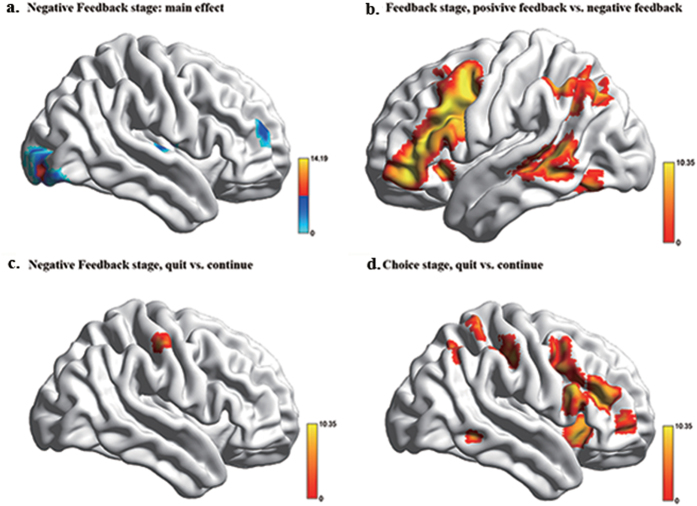
Whole-brain analysis results. (**a**) Neuroanatomical location of brain regions during the feedback stage. (**b**,**c**) Brain regions exhibiting significant increased activation in the contrast between positive and negative feedback (**b**) and between quitting and continuing with a stimulus set (**c**) during the feedback stage, and the latter contrast during the choice stage (**d**). FWE, *p* < 0.05 corrected and >10 voxels.

To investigate which brain regions showed activity changes associated with negative feedback in the task, we first performed a whole-brain analyses on fMRI data collected during the feedback stage. Since there were too few trials where subjects had more than 4 failures to perform an effective analysis of fMRI data we only analyzed neural changes following 1, 2, 3 and 4+ failures. This analysis revealed that a number of middle and superior frontal, temporal and occipital cortical regions and the ventral striatum showed increased activity whereas medial and dorsolateral frontal regions showed decreased activity. These neural activity changes were significantly associated with the number of times negative feedback was received (see Table 1 and Fig. 2a). A number of frontal regions also showed activation differences in the contrast between positive and negative feedback (Table 1 and Fig. 2b). Greater activation changes during the receipt of negative feedback that were associated with the subsequent decision to quit as opposed to continue only occurred in dACC/MPFC (Table 1 and Fig. 2c), whereas in other frontal and temporal regions greater activation changes associated with the decision to quit rather than to continue only occurred during the actual choice stage (Table 1 and Fig. 2d).

### Neural correlates of number of negative feedbacks received and quitting rate

To explore how activity changes in the different brain regions related to the number of times negative feedback was received and quitting rate, we used the same quadratic polynomial function as for analysis of the behavioral data. Brain activations were extracted from 8 mm diameter spheres in the following ROIs: MPFC (0, 47, 21), bilateral dACC (0, 26, 29), left insula (−36, 23, 0), right DLPFC (39, 53, 19), left and right VStr (−24, 2, 7/27, −4, 10), left PCC (−9, −22, 43), right precuneus (12, −49, 55), right amygdala (23, −11, −27), left middle temporal gyrus (MTG, −51, −13, −14) and right TPJ (66, −49, 1) .

Repeated-measures ANOVA was used to test whether the parameter estimates of each target region were significantly different for each of the four frequencies of negative feedback (i.e. whether the pattern of each target region was similar to that observed in the behavioral results). The results showed there were main effects of negative feedback in dACC, PCC and TPJ [*F*_(*3,76*)_ = 3.19, *p* = 0.028; *F*_(*3,76*)_ = 4.24, *p* = 0.008; *F*_(*3,76*)_=5.02, *p* = 0.003), i.e. they showed the same trend as the behavioral results. Post-hoc, Bonferroni-corrected analysis results showed that the parameter estimate for the first negative feedback was significantly different from the second (*p* = 0.048), the third (*p* = 0.01) and fourth (*p* = 0.005) respectively in dACC (*M1* = −0.095, *SD1* = 0.34, *M2* = 0.045, *SD2* = 0.43, *M3* = 0.21, *SD3 *= 0.39, *M4* = 0.26, *SD4* = 0.43). For PCC (*M1* = −0.071, *SD1* = 0.25, *M2* = −0.035, *SD2* = 0.25, *M3* = −0.15, *SD3* = 0.12, *M4* = −0.24, *SD4* = 0.16), there were no significant differences between the first, second and third feedbacks (*p* > 0.05) but there was for the fourth one (*p* = 0.037). For the TPJ (*M1* = −0.063, *SD1* = 0.26, *M2* = −0.21, *SD2* = 0.28, *M3* = −0.32, *SD3* = 0.31, *M4* = −0.39, *SD4* = 0.29) there were no differences between the first two times of negative feedback (*p* = 0.119) but there were between the first and the third and fourth times (*p* = 0.007, *p* = 0.005, see Fig. S2a,c,d).

**Figure 3:**
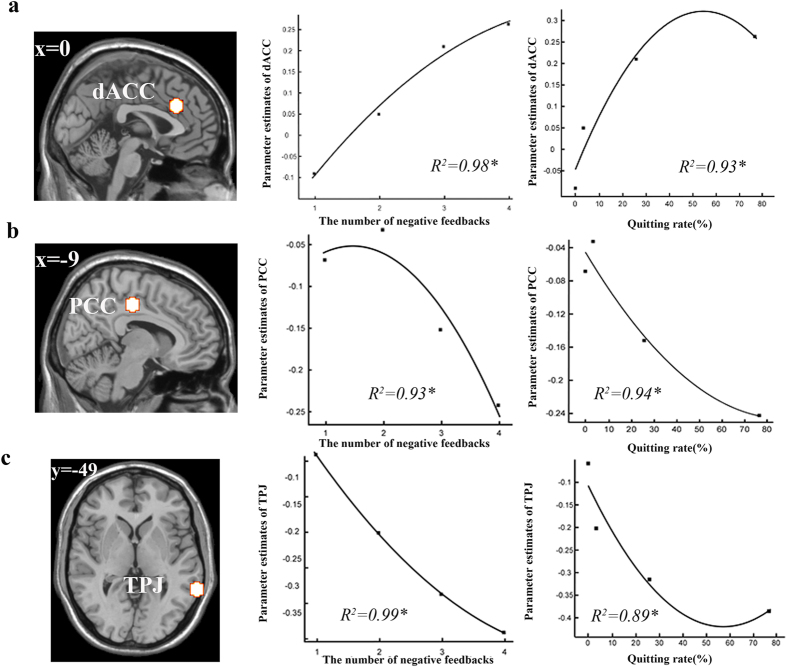
ROI results. (**a**–**c**) Brain activations were extracted from an 8 mm diameter spherical dACC, PCC, TPJ ROIs. The relationship between parameter estimates of dACC (**a**), PCC (**b**), TPJ (**c**) and the number of negative feedbacks or quitting rate is a quadratic polynomial function.

We also explored how activity changes in dACC, PCC and TPJ related to the number of times negative feedback was received and quitting rate. First, we found that parameter estimates of dACC, PCC and TPJ fitted the same quadratic polynomial function (*R*^*2*^ = 0.98, *R*^*2*^ = 0.93, *R*^*2*^ = 0.99, *p* < 0.05 in all cases). We next explored the relationship between parameter estimates of dACC, PCC and TPJ and quitting rate and obtained similar results (*R*^*2*^ = 0.93, *R*^*2*^ = 0.94, *R*^*2*^ = 0.89, *p* < 0.05 in all cases, Fig. 3a–c).

For completeness we also investigated relationships between activity changes and number of feedbacks received and quitting rate using a linear regression. Results were broadly similar to those obtained using a quadratic polynomial function (see Supplementary information and Fig. S2b).

### Interactions between neural systems

Having identified dACC, PCC and TPJ as the main regions associated with the number of times negative feedback was received, we next aimed to determine whether any of their functional connections showed a similar association. We therefore performed a set of generalized form of context-dependent psychophysiological interactions (gPPI) analyses using the dACC, PCC and TPJ as seed ROIs.

**Figure 4:**
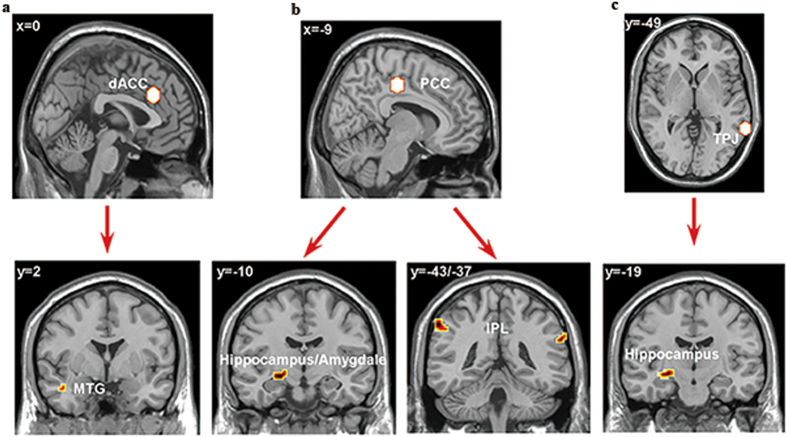
gPPI results. (**a**) The dACC seed had increased functional connectivity with MTG for the f3 + f4 condition. (**b**) The PCC seed had increased functional connectivity with bilateral IPL and hippocampus/amygdala for f3 + f4 condition. (**c**) The TPJ seed had increased connectivity with hippocampus for f3 + f4 condition (*p* < 0.001, small volume corrected).

We contrasted the level of functional connectivity during the feedback stage with different numbers of times negative feedback was received. The analyses showed that there was significantly increased functional connectivity after ≥3 failures (f3 + f4) versus 1–2 failures (f1 + f2) for dACC-MTG (−45, 2, −26), TPJ-hippocampus (−30, −19, −11), PCC-hippocampus/amygdala (−21, −10, −11) and PCC-inferior parietal lobule (IPL, −57, −43, 43/69, −37, 28) (all small volume corrected, *p* < 0.001; see Fig. 4).

## Discussion

Using fMRI and a novel decision task paradigm the current study has elucidated how repeated negative feedback (failure) affects people’s subsequent decision-making behavior. Our findings show that the more times subjects experience failure the more likely they are to give up, with three failures being the key threshold for subjects to start quitting and the majority of them doing so after a fourth failure. The brain areas most closely associated with the number of times negative feedback was received, and predictive of whether subjects gave up, were the dACC in particular but also the PCC and TPJ.

In the current study, we found activity and functional connectivity changes in the dACC, PCC and TPJ were associated with receiving negative feedback, which is consistent with previous studies^18^,^27^. Neural activity changes have also been described which are related to this effect of negative feedback, such as encoding the value of outcomes^28^, expectation of the value of the available actions^29^ and monitoring or evaluation of previous actions^30^. These changes have been found in diverse brain regions including the ACC, MPFC, DLPFC, amygdala, and striatum^18^. Although behavioral decisions are likely to depend on information computed in this network of brain regions, it is not yet known which components are of specific importance for decision making which guides the selection of action^18^. Our finding from the contrast between the decision to quit or continue that the ACC is activated during receipt of negative feedback, rather than during the actual choice stage, suggests it is particularly involved in error detection and monitoring of behavioral responses.

Our behavioral results showed that the more times subjects experienced failure the more likely they were to give up. Three failures appeared to be point at which there was a clear increase in quitting behavior, indicating that this was a key threshold. However, the greatest proportion of subjects decided to quit after four failures. The relationship between the number of times negative feedback was received and quitting rate fitted a quadratic polynomial function. Based on this we extracted the parameter estimates from regions of interest that fitted the same function. The “three strikes” phenomenon is well known and relates to the “hot hand” belief 24,25 . However, in all previous studies it is clear that the paradigms used resulted in subjects having increased expectation of success after each failure. In our study, we also asked subjects to report their confidence level after each round and this showed that their confidence in success decreased with successive failures and confidence level was negatively correlated with the number of negative feedbacks received (see Supplementary Information and Fig. S1). Thus in this paradigm subjects had a progressively higher expectation of failure (not success) after each failed trial.

In our study changes in BOLD activity were only found during the feedback stage and not during the choice one and thus primarily reflect changes occurring following decision making. We also found that dACC activation could predict behavioral choice when subjects received a second and third negative feedback (see Supplementary Information and Fig. S2b). The ACC, as a component of cognitive control network (CCN), engages multiple processes, including task switching, response inhibition, error detection, response conflict and working memory^31^,^32^,^33^,^34^,^35^,^36^. On the other hand, the ACC may play a central role in decision making due to its involvement in both learning and using extended action-outcome histories to optimize choice behavior^3^,^37^,^38^. Reinforcement learning theory postulates that the ACC is involved in learning after obtaining an outcome that is worse than expected, i.e., a reward prediction error^38^,^39^. Importantly, in our current study activity in dACC could reliably predict whether subjects continued or gave up. The ACC has also been implicated in reward-action associations, average expected values and negative reward prediction errors^3^,^40^,^41^,^42^,^43^. It is likely that the key role of the ACC in these processes is due in part to its position within the reward system, and also on its use of outcome information for action value adjustments and behavioral regulation^44^. In humans, ACC lesions promote response slowing and variability^45^ but the ability to learn from feedback is spared^46^,^47^. Thus, the function we can attribute to the dACC activity changes we have found may be not only to evaluate feedback but also to participate in monitoring the different steps of the task at hand to optimize action adaptation and valuation^44^.

The PCC is also an important region linking reward processing, attention, memory and motor control systems, and mediates the integration of variables such as reward, uncertainty, errors and option switching across multiple trials^48^. Recent nonhuman primate work provides evidence for a more active role in the control of cognition through signaling an environmental change and the need to alter behavior^20^. In this probabilistically rewarded choice task, monkeys’ behavior followed a win-stay or lose-shift heuristic. After choosing the risky option, monkeys were more likely to choose it again if they received a larger reward but switched to the safe option if they received a smaller one. Firing rates of PCC neurons were correspondingly higher following smaller rewards than large ones, and variability in responses predicted the likelihood monkeys would switch their choice on the next trial^20^. Thus the PCC, as well as dACC, is a key node responsible for environmental change detection and subsequent alterations in behavioral strategy.

The right TPJ is also activated by unexpected stimulus events of behavioral relevance^19^. The TPJ is involved in stimulus-driven representation of task-relevant information that can be used to engage an appropriate behavioral response^19^,^49^. Although implicated in stimulus detection, the TPJ serves critical social cognitive and regulatory roles subserving higher order cognitive processes (e.g. perspective taking, mentalizing, theory of mind)^50^,^51^. In summary therefore, dACC, PCC and TPJ are all associated with valuation, reward learning and decision-making functions.

In addition, gPPI results showed increased dACC-MTG/TPJ -Hippocampus/PCC-Hippocampus/PCC-IPL functional connectivity after three or more failures compared to after only one or two. Attention-demanding, goal-directed behavior is mediated not only by distributed patterns of cerebral activation but, remarkably, also by concurrent suppression of activity in a distinct set of brain areas, such as fronto-parietal circuits (dACC/PCC/IPL/TPJ)^49^,^52^,^53^. The PCC forms strong, reciprocal connections with the medial temporal lobe, especially the parahippocampal gyrus, which is important for associative learning and episodic memory^20^. We also observed greater functional connectivity between right TPJ and a number of attentional and decision-making areas, including the right inferior frontal gyrus, MPFC and parahippocampal gyrus^19^.

Von der Gablentz *et al.*^54^ used a modified Eriksen–Flanker task that required the participants to derive the correct stimulus–response association based on a feedback given after each flanker stimulus. Participants had to continuously monitor and adapt their performance as the stimulus–response contingency switched. The results showed that the switch was associated with activation of the precuneus, the cingulate cortex, the insula and brainstem regions. This brainstem system appears to interact with the cortical network and seems to be essential for performance monitoring and behavioral adaptation. One key difference is that in the reversal paradigm, stimulus-response contingencies were switched. In our study, we have used a visual shape discrimination task with computer-controlled feedback, where the subject could either decide to continue (try again) or abort (quit and try a new discrimination) after failing on a trial. We focused on the critical number of sequential trials on which subjects failed before deciding to quit and try a new stimulus set. The brain areas most closely associated with the number of times negative feedback was received, and predictive of whether subjects give up, are the dACC, PCC and TPJ, especially the dACC. It appears that in the human brain computation of both error monitoring and reward outcome to guide decision making is associated with integrated changes in frontal, parietal, temporal and limbic circuitry. While, the two tasks have in common that the same stimulus display is shown repeatedly, so that participants can also effectively “try again” in the reversal task, an important difference is that in the reversal task repeated failures are not guaranteed to occur in immediate succession.

According to principles of reinforcement learning, our results can be interpreted as a reward prediction error signal sent from posterior regions (PCC, TPJ) to more anterior circuitry (dACC) involved in behavioral adaptation, with the dACC monitoring how behavior is adjusted in the future^7^,^23^,^38^,^47^. However, some limitations in our current study need to be taken into account. A major source of difficulty in interpreting our findings concerns the nature of stay/switch decision making. In our paradigm making the decision to switch was easier than deciding to continue and uncertainty has a clear influence on decision making (1–2 feedbacks = my decision to stay is clear, 3–4 feedbacks = greater uncertainty). Thus it is difficult to determine from our paradigm and results what influence uncertainty may have played. In addition, the paradigm deliberately made it hard for subjects to learn anything about the stimuli in order to investigate pure negative feedback effects and exclude learning, and increasing expectations of success, as potential confounding factors. As such the paradigm may be less ecologically relevant compared with one in which a behavioral strategy can at least implicitly be learned and adapted to in accordance with feedback received.

In summary, the present study has provided new insights into both patterns of behavior and changes in associated neural circuitry during the experience of repeated failure in a visual discrimination task. Behaviorally, the more times subjects experienced failure the more likely they were to give up, with three failures being the key threshold for starting to adopt quitting and the majority of subjects actually quitting after receiving a fourth negative feedback. Changes in the activity and functional connectivity of dACC, PCC and TPJ were particularly associated with the number of previous failures and also predictive of subjects giving up. Both the pattern of behavior and that of activity changes in these three regions fitted a quadratic polynomial regression model. The current findings therefore provide new evidence for an association between the experience of repeated failures and adaptive changes in decision making. It appears that in the human brain the decision to quit after three/four failures is associated with integrated changes in frontal, parietal, temporal and limbic circuitry, which serve to compute both error monitoring and reward outcome to guide decision making.

## Methods

### Participants

20 healthy right-handed undergraduate Han Chinese subjects with no history of neurological or psychiatric disorder (*M*_*age*_ = 22.0, *SD* = 2.07; 9 males) were recruited to participate in the experiment. All subjects provided written informed consent and were paid according to their performance in the experiment. The study was approved by the ethics committee of Southwest University (China) and the Institutional Human Participants Review Board of the Southwest University Imaging Center for Brain Research. The methods were carried out in accordance with approved guidelines.

### Experimental procedures and stimuli

**Figure 5:**
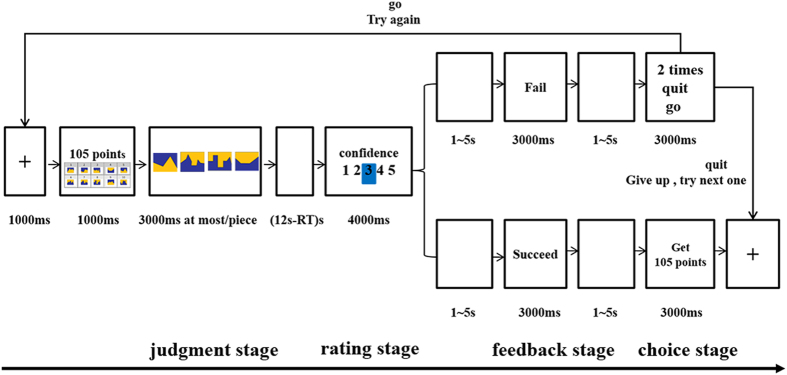
Visual shape discrimination task. Each trial in the task started with, a fixation point displayed in the center of the screen for 1000 ms. Next, ten pictures (each picture was divided into two colored areas) in a specific stimulus set, and the potential reward value, were shown for 1000 ms. Subjects were required to make judgments on which of the two colored areas was larger for four pictures, which were randomly selected from the ten pictures in the stimulus set (judgment stage). For each picture, subjects were given a maximum of 3 s to make their judgment and after the last decision was made the display screen went blank for the remainder of the 12 s. After subjects had made all decisions they gave their confidence level on their accuracy on a scale of 1 to 5 (rating stage – 4s). Subjects were told that if they accurately identified which colored part was larger in at least 3/4 stimuli then they would be informed on the screen after 3s (1–5 s jittered interval) that they had passed, otherwise they would be told that they had failed (feedback stage). After a further 3s interval (1–5s jittered) if the subjects passed they were informed they had gained the points on offer (3s) and then moved on to the start of another stimulus set. If subjects failed they were informed that they had won 0 points and how many times they had failed on that specific stimulus set (number displayed on the top of the screen). Subjects were then given the choice to either try again with the same stimulus set or to quit and try a new one (choice stage).

Before the fMRI session, subjects participated in 5 training trials after being given detailed instructions. In the task, subjects were asked to play a game where they were required to determine from ten stimuli comprising two different colored areas which had the largest overall area and with varying degrees of difficulty. After a decision round involving four (3 hard and 1 easy) out of the ten possible stimuli (8 hard and 2 easy) the subjects received feedback (succeed or fail) and when the feedback was negative they were given an alternative to either continue with same stimulus set again or to quit and try a new stimulus set. In this task, there are actually 112 different permutations for each stimulus set (i.e. C^1^_2_*C^3^_8_ = 2*8*7*6/3*2 = 2*56 = 112). The subject is therefore unable to reach the conclusion that they cannot solve the condition after only 2 or 3 failures. Subjects could continue to try each stimulus set as many times as they wanted and so could receive as many negative feedbacks as they were prepared to accept. The monetary compensation subjects received depended on the total number of points they gained from stimulus sets where they successfully passed the discrimination test (Money (RMB) = points/1000). The visual discrimination task paradigm was written in E-prime 2.0 (http://www.pstnet.com/eprime.cfm, Psychology Software Tools, USA) and the specific task flow is shown in Fig. 5.

One hundred and sixty pictures created by Computer Aided Design software (CAD) were used as stimuli in the perception task. Each picture (18 mm*10 mm) was subdivided into two irregular parts which were randomly colored either orange or blue and separated by a jagged line. To help avoid the subjects realizing that they were sometimes receiving false feedback and to keep them motivated by making sure the task was not too difficult, we used two different levels of difficulty (‘easy’ or ‘hard’), although subjects were not informed about this before performing the task. Stimuli were divided into those which were ‘easy’ (32 pictures where subjects achieved 95% ± 5% accuracy and the area size difference was >30%) or ‘hard’ (128 pictures where subjects achieved 55% ± 5% and the area difference was <15%) based on the findings of a prior experiment. Stimuli were divided into those which were ‘easy’ (32 pictures where subjects achieved 95% ± 5% accuracy and the area size difference was >30%) or ‘hard’ (128 pictures where subjects achieved 55% ± 5% and the area difference was <15%) based on the findings of a prior experiment. In a pilot study, 22 healthy right-handed undergraduate Han Chinese subjects (11 females and 11 males, M_age_ = 20.7 years old) performed the study in order to assess the difficulty of the stimuli. Each picture was presented four times, making 640 pictures in total. In addition, subjects received false feedback (succeed or fail) calculated in terms of probability using permutation and combination functions (see Table 2). For example, where the probability of a correct feedback was (C^3^_4_ + C^4^_4_)* (1/2)^4^ = 5/16 = 31% then the number of times false correct feedback was given was 31%*64 = 20 (Permutation A). Where the subjects were wrong on the first occasion, and then succeeded on the next attempt, the probability was (C^0^_4_ + C^1^_4_ + C^2^_4_)*(1/2)^4^ *(C^3^_4_ + C^4^_4_)* (1/2)^4^ = 11/16 *5/16 = 21%. Thus the number of times false feedback was given for this permutation was 21%*64 = 13 (Permutation B), and so on for Permutations C, D, E, F and G. Using this approach we could systematically manipulate the number of failures each subject experienced and no subjects reported being aware of receiving false feedback.

Each trial had four stages: judgment, rating, feedback and choice. At the beginning of the trial a fixation cross was displayed in the center of the screen for 1000 ms. Next, the ten pictures in a specific stimulus set were shown (easy or hard discrimination difficulty) and the potential reward value (100–120 points in increments of 5) for 1000 ms. Here, the reward was just to motivate subjects to take part in the experiment seriously. The range of reward values was very small (100–120) and was varied slightly just to help increase subjects’ attention. Subjects were then required to make judgments on a random selection of four out of the ten pictures in the stimulus set for which color had the largest overall area (judgment stage). In each case the subjects were given a maximum of 3 s to make their judgment and after the last decision was made the display screen went blank for the remainder of the 12 s. After subjects had made all their decisions they gave their confidence level on their accuracy on a scale of 1 (no confidence) to 5 (very high confidence) (rating stage – 4s). Subjects were told that if they accurately identified which colored part was larger in at least 3/4 stimuli (i.e. 3/4 or 4/4) then they would be informed on the screen after 3s (1–5 s jittered interval) that they had passed, otherwise they would be told that they had failed (feedback stage). In fact, as described above, all feedback given was computer controlled and calculated in terms of probability, making it hard for subjects to learn anything about the picture sets from either their successes or failures. After a further 3s interval (1–5s jittered), if the subjects had passed they were informed that they had gained the points on offer (3s) and then moved on to the start of another stimulus set. If the subjects had failed they were informed that they had won 0 points and how many times they had failed on that specific stimulus set (number displayed on the top of the screen). Subjects were then given the choice to either try again with the same stimulus set or to quit and try a new one (choice stage). In all cases where decisions were made the response time to press the button was recorded. Overall the task included 160 different stimulus sets presented in a random sequence of three difficult and one easy. Since we wanted to reveal subjects’ normal decision making strategies no limit was placed on the number of times they could fail and choose to continue with a specific stimulus set. Mean monetary compensation subjects received was 60 RMB.

### fMRI data acquisition

During each fMRI scan an Echo-Planar imaging (EPI) sequence was used for data collection, and T2*-weighted images were recorded per run using a Siemens TRIO 3.0T MRI scanner [repetition time (TR) = 2000 ms; echo time (TE) = 30 ms; flip angle = 90°; FoV = 220 × 220 mm^2^; matrix size = 64 × 64; 32 interleaved 3 mm-thick slices; in-plane resolution = 3.4 × 3.4 mm^2^; inter slice skip = 0.99 mm]. Additionally, anatomical images (256 × 256 × 176) with 1 × 1 × 1 mm^3^ resolution were obtained by a T1-weighted three-dimensional magnetization prepared rapid gradient echo (MPRAGE) sequence (inversion time = 900 ms; TR = 1900 ms; TE = 2.52 ms; flip angle = 9°).

### fMRI data analyses

Statistical analyses were performed using the general linear model in SPM8^55^. Slice timing was used to correct slice order, the data was realigned to estimate and modify the six parameters of head movement and the first three images were discarded to achieve magnet-steady images. These images were then normalized to MNI space in 3 × 3 × 3 mm^3^ voxel sizes. The normalized data were spatially smoothed with a Gaussian kernel; the full width at half maximum (FWHM) was specified as 8 × 8 × 8 mm^3^. After pre-processing, the ten regressors from each run [i.e., judgment stage (stimulus/judgment), rating stage (rating), feedback stage (correct, 1, 2, 3, 4 or more failures – correct, f1/f2/f3/f4), choice stage (continue/quit)] were modeled to create the design matrix. The regressors were then convolved with the Canonical Hemodynamic Response Function and the six realignment parameters for each subject were also included as confounding factors.

### Whole-brain analyses

Our results primarily relate to feedback processing We investigated brain regions whose activation was associated with the process of negative feedback using one-sample t-tests. Moreover, we also investigated the contrast between positive (correct and get points) and negative feedback (f1 + f2 + f3 + f4). In addition, the contrast between the decision to stay or shift after negative feedback during both the negative feedback and choice stages was conducted. The image threshold for fMRI data significance was set to, *p* < 0.05 FWE corrected, with clusters of ≥10 contiguous voxels.

### Region of interest analyses (ROI)

We used the MarsBar toolbox^56^ for use with SPM8 to perform ROI analyses to further characterize patterns of activation which matched behavioral decision making performance. Brain activation was extracted from 8 mm diameter spheres centered on co-ordinates identified in previous research. ROIs included the MPFC (0, 47, 21), dACC (0, 26, 29), insula (−36, 23, 0), amygdala (23, −11, −27), DLPFC (39, 53, 19), VStr (−24, 2, 7/27, −4, 10), PCC (−9, −22, 43), precuneus (12, −49, 55), TPJ(66, −49, 1) and MTG (−51, −13, −14)^6^,^18^,^57^ using MarsBar software. Repeated-measures ANOVA was used to test whether the parameter estimates for each of the target regions were significantly different across the four frequencies (1–4 times) of negative feedback. Quadratic polynomial function analysis was conducted to explore the relationship between ROI parameter estimates and quitting rate at different frequencies of negative feedback.

### Functional connectivity analyses

Based on behavioral results, we could infer that subjects tended to continue with the same stimulus set after up to 2 failures (f1 + f2) but would quit after ≥3 failures (f3 + f4). Functional connectivity strengths were then analyzed to investigate changes between regions associated with behavioral choice (i.e. continue or quit). We measured functional connectivity using gPPI analysis^55^,^58^,^59^. For the gPPI analysis, we extracted the de-convolved time-course of each seed region (dACC, PCC, TPJ) in each subject, based on an 8 mm radius sphere centered on the peak-activation voxel from the ROI. We calculated the product of this activation time-course and the vector of the psychological variable of interest to create the psychophysiological interaction term. New SPMs were computed for each subject, including the interaction term, the physiological variable (i.e. the ROI activation time course) and the psychological variable as regressors. We then identified areas where activation was predicted by the psychophysiological interaction term, with ROI activity and the psychological regressor treated as confound variables. These analyses were carried out separately for both f1 + f2 and f3 + f4 conditions. Individual PPI SPMs were entered into a random-effects group analysis contrasting connectivity patterns between f1 + f2 and f3 + f4 conditions with a one-sample t-test, threshold at *p* < 0.001, small volume corrected, with a minimum cluster size of 10 voxels.

## Additional Information

**How to cite this article**: Zhao, W. *et al.* Neural circuitry involved in quitting after repeated failures: role of the cingulate and temporal parietal junction. *Sci. Rep.* **6**, 24713; doi: 10.1038/srep24713 (2016).

## Supplementary Material

Supplementary Information

## Figures and Tables

**Table 1:** The table shows anatomical regions with significant activation changes, their laterality, and BA, Brodmann’s areas.

Brain Region	BA	No. Voxels	Peak t-value	x	Y	z
**Negative feedback** > **baseline**						
R. Superior Frontal Gyrus	10	19	7.47	39	53	19
R. Ventral Striatum		52	8.81	27	−4	10
R. Ventral Striatum		27	8.11	−24	2	7
R. Superior Temporal Gyrus	41/6	28	7.38	57	−19	7
R. Inferior Occipital Gyrus	18/19	142	10.50	30	−94	−11
**Baseline** > **Negative feedback**						
R. Medial Prefrontal Cortex	9/6	17	7.23	3	44	37
L. Dorsolateral Frontal Cortex	45	67	8.79	−45	29	7
L. Inferior Frontal Gyrus	13/47	22	7.55	−30	23	−8
R. Inferior Frontal Gyrus	13	11	7.44	42	26	10
R. Inferior Frontal Gyrus	47	35	9.22	27	26	−11
R. Thalamus		136	9.20	6	−16	−2
**Positive feedback** > **Negative feedback**						
R. Middle Frontal Gyrus	46/9/47	1638	19.61	51	26	31
Inferior Frontal Gyrus						
Ventral Striatum						
R. Medial Frontal Gyrus	8	18	7.19	3	32	46
L. Middle Frontal Gyrus	9/46	1039	14.34	−42	8	46
Inferior Frontal Gyrus						
L. Inferior Frontal Gyrus	13/47	45	9.79	−33	23	−5
L. Anterior Cingulate Cortex	32/24	73	7.89	−3	41	−2
R. Posterior Cingulate Cortex	31/23	100	9.55	6	−31	34
R. Precuneus	7	29	7.87	12	−67	40
L. Superior Temporal Gyrus	22	310	9.61	−57	−46	7
R. Middle Temporal Gyrus	21	191	9.16	54	−46	−11
L. Inferior Parietal Lobule	40/7	332	9.01	−42	−67	43
Superior Parietal Lobule						
R. Inferior Parietal Lobule	40/7	377	9.05	54	−58	43
Superior Parietal Lobule						
**Feedback stage: Quit** > **continue**						
R. Medial Frontal Gyrus	32/9	33	7.41	9	26	34
Anterior Cingulate Cortex						
**Choice stage: Quit** > **continue**						
R. Inferior Frontal Gyrus	47	97	9.32	36	20	−8
R. Middle Frontal Gyrus	10	35	8.84	39	50	1
R. Inferior Frontal Gyrus	9	224	10.11	51	14	25
R. Middle Frontal Gyru	46	142	8.84	48	32	22
L. Insula	13	29	7.31	−33	17	4
R. Middle Temporal Gyrus		13	7.06	54	−46	−5
R. Inferior Parietal Lobule	7	60	7.05	33	−61	40

Areas of brain activation for negative feedback (MNI coordinates).

**Table 2:** The number of task outcome permutations. Every permutation type was included in the sequence and followed a random probability principle.

Permutation type	1	2	3	4	5	6	7	8	9	10	11	12	No. of false positive feedbacks
G	X	X	X	X	X	X	X	X	X	X	X	X	7
F	X	X	X	X	X	X	X	X	X	√			6
E	X	X	X	X	X	X	X	√					6
D	X	X	X	X	X	√							6
C	X	X	X	√									6
B	X	√											13
A	√												20

“X” mean negative feedback presented.

“√” mean correct.

The random possibility of Permutation type A is 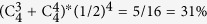.

The random possibility of Permutation type B is 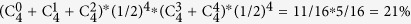.

The random possibility of Permutation type C is 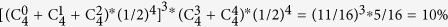.

## References

[b1] Morita K., Morishima M., Sakai K. & Kawaguchi Y. Reinforcement learning: computing the temporal difference of values via distinct corticostriatal pathways. Trends Neurosci. 35, 457–467 (2012). 22658226 10.1016/j.tins.2012.04.009

[b2] Seo H. & Lee D. Temporal filtering of reward signals in the dorsal anterior cingulate cortex during a mixed-strategy game. J. Neurosci. 27, 8366–8377 (2007). 17670983 10.1523/JNEUROSCI.2369-07.2007 PMC2413179

[b3] Kennerley S. W., Walton M. E., Behrens T. E. J., Buckley M. J. & Rushworth M. F. S. Optimal decision making and the anterior cingulate cortex. Nat. Neurosci. 9, 940–947 (2006). 16783368 10.1038/nn1724

[b4] Nassar M. R., Wilson R. C., Heasly B. & Gold J. I. An approximately Bayesian delta-rule model explains the dynamics of belief updating in a changing environment. J. Neurosci. 30, 12366–12378 (2010). 20844132 10.1523/JNEUROSCI.0822-10.2010 PMC2945906

[b5] Nassar M. R. *et al.* Rational regulation of learning dynamics by pupil-linked arousal systems. Nat. Neurosci. 15, 1040–1046 (2012). 22660479 10.1038/nn.3130 PMC3386464

[b6] Amiez C., Sallet J., Procyk E. & Petrides M. Modulation of feedback related activity in the rostral anterior cingulate cortex during trial and error exploration. Neuroimage 63, 1078–1090 (2012). 22732558 10.1016/j.neuroimage.2012.06.023

[b7] Ridderinkhof K. R., van den Wildenberg W. P. M., Segalowitz S. J. & Carter C. S. Neurocognitive mechanisms of cognitive control: the role of prefrontal cortex in action selection, response inhibition, performance monitoring, and reward-based learning. Brain Cogn. 56, 129–140 (2004). 15518930 10.1016/j.bandc.2004.09.016

[b8] Bayer H. M. & Glimcher P. W. Midbrain dopamine neurons encode a quantitative reward prediction error signal. Neuron 47, 129–141 (2005). 15996553 10.1016/j.neuron.2005.05.020 PMC1564381

[b9] Hollon N. G., Soden M. E. & Wanat M. J. Dopaminergic prediction errors persevere in the nucleus accumbens core during negative reinforcement. J. Neurosci. 33, 3253–3255 (2013). 23426653 10.1523/JNEUROSCI.5762-12.2013 PMC3608139

[b10] Santesso D. L. *et al.* Individual differences in reinforcement learning: behavioral, electrophysiological, and neuroimaging correlates. Neuroimage 42, 807–816 (2008). 18595740 10.1016/j.neuroimage.2008.05.032 PMC2548326

[b11] Samson R., Frank M. & Fellous J. M. Computational models of reinforcement learning: the role of dopamine as a reward signal. Cogn. Neurodyn. 4, 91–105 (2010). 21629583 10.1007/s11571-010-9109-x PMC2866366

[b12] Law C. T. & Gold J. I. Reinforcement learning can account for associative and perceptual learning on a visual-decision task. Nat. Neurosci. 12, 655–663 (2009). 19377473 10.1038/nn.2304 PMC2674144

[b13] Hayden B. Y., Heilbronner S. R., Pearson J. M. & Platt M. L. Surprise signals in anterior cingulate cortex: neuronal encoding of unsigned reward prediction errors driving adjustment in behavior. J. Neurosci. 31, 4178–4187 (2011). 21411658 10.1523/JNEUROSCI.4652-10.2011 PMC3070460

[b14] Matsumoto M. & Hikosaka O. Lateral habenula as a source of negative reward signals in dopamine neurons. Nature 447, 1111–1115 (2007). 17522629 10.1038/nature05860

[b15] Rushworth M. F. S., Buckley M. J., Behrens T. E. J., Walton M. E. & Bannerman D. M. Functional organization of the medial frontal cortex. Curr. Opin. Neurobiol. 17, 220–227 (2007). 17350820 10.1016/j.conb.2007.03.001

[b16] Lee D., Seo H. & Jung M. W. Neural basis of reinforcement learning and decision making. Annu. Rev. Neurosci. 35, 287–308 (2012). 22462543 10.1146/annurev-neuro-062111-150512 PMC3490621

[b17] Niv Y. Reinforcement learning in the brain. J. Math. Psychol. 53, 139–154 (2009).

[b18] Hampton A. N. & O’Doherty J. P. Decoding the neural substrates of reward-related decision making with functional MRI. Proc. Natl. Acad. Sci. 104, 1377–1382 (2007). 17227855 10.1073/pnas.0606297104 PMC1783089

[b19] Geng J. J. & Mangun G. R. Right temporoparietal junction activation by a salient contextual cue facilitates target discrimination. Neuroimage 54, 594–601 (2011). 20728548 10.1016/j.neuroimage.2010.08.025 PMC2993878

[b20] Pearson J. M., Heilbronner S. R., Barack D. L., Hayden B. Y. & Platt M. L. Posterior cingulate cortex: adapting behavior to a changing world. Trends Cogn. Sci. 15, 143–151 (2011). 21420893 10.1016/j.tics.2011.02.002 PMC3070780

[b21] van den Bos W., Talwar A. & McClure S. M. Neural Correlates of Reinforcement Learning and Social Preferences in Competitive Bidding. J. Neurosci. 33, 2137–2146 (2013). 23365249 10.1523/JNEUROSCI.3095-12.2013 PMC6619103

[b22] Cohen M. X. & Ranganath C. Reinforcement learning signals predict future decisions. J. Neurosci. 27, 371–378 (2007). 17215398 10.1523/JNEUROSCI.4421-06.2007 PMC6672075

[b23] Cohen M. X., Wilmes K. A. & van de Vijver I. Cortical electrophysiological network dynamics of feedback learning. Trends Cogn. Sci. 15, 558–566 (2011). 22078930 10.1016/j.tics.2011.10.004

[b24] Carlson K. A. & Shu S. B. The rule of three: How the third event signals the emergence of a streak. Organ. Behav. Hum. Decis. Process. 104, 113–121 (2007).

[b25] Raab M., Gula B. & Gigerenzer G. The hot hand exists in volleyball and is used for allocation decisions. J. Exp. Psychol. Appl. 18, 81 (2012). 22004053 10.1037/a0025951

[b26] Fleck M. S., Daselaar S. M., Dobbins I. G. & Cabeza R. Role of prefrontal and anterior cingulate regions in decision-making processes shared by memory and nonmemory tasks. Cereb. Cortex 16, 1623–1630 (2006). 16400154 10.1093/cercor/bhj097

[b27] Stern E. R., Gonzalez R., Welsh R. C. & Taylor S. F. Updating beliefs for a decision: neural correlates of uncertainty and underconfidence. J. Neurosci. 30, 8032–8041 (2010). 20534851 10.1523/JNEUROSCI.4729-09.2010 PMC2896864

[b28] O’Doherty J., Kringelbach M. L., Rolls E. T., Hornak J. & Andrews C. Abstract reward and punishment representations in the human orbitofrontal cortex. Nat. Neurosci. 4, 95–102 (2001). 11135651 10.1038/82959

[b29] Platt M. L. & Glimcher P. W. Neural correlates of decision variables in parietal cortex. Nature 400, 233–238 (1999). 10421364 10.1038/22268

[b30] Cohen J. D., Botvinick M. & Carter C. S. Anterior cingulate and prefrontal cortex: who’s in control? Nat. Neurosci. 3, 421–423 (2000). 10769376 10.1038/74783

[b31] Rushworth M. F. S., Noonan M. P., Boorman E. D., Walton M. E. & Behrens T. E. Frontal cortex and reward-guided learning and decision-making. Neuron 70, 1054–1069 (2011). 21689594 10.1016/j.neuron.2011.05.014

[b32] Gläscher J. *et al.* Lesion mapping of cognitive control and value-based decision making in the prefrontal cortex. Proc. Natl. Acad. Sci. 109, 14681–14686 (2012). 22908286 10.1073/pnas.1206608109 PMC3437894

[b33] Dosenbach N. U. *et al.* Distinct brain networks for adaptive and stable task control in humans. Proc. Natl. Acad. Sci. 104, 11073–11078 (2007). 17576922 10.1073/pnas.0704320104 PMC1904171

[b34] Alexopoulos G. S. *et al.* Functional connectivity in the cognitive control network and the default mode network in late-life depression. J. Affect. Disord. 139, 56–65 (2012). 22425432 10.1016/j.jad.2011.12.002 PMC3340472

[b35] Sheline Y. I., Price J. L., Yan Z. & Mintun M. A. Resting-state functional MRI in depression unmasks increased connectivity between networks via the dorsal nexus. Proc. Natl. Acad. Sci. 107, 11020–11025 (2010). 20534464 10.1073/pnas.1000446107 PMC2890754

[b36] Bosch O. G. *et al.* Sleep deprivation increases dorsal nexus connectivity to the dorsolateral prefrontal cortex in humans. Proc. Natl. Acad. Sci. 110, 19597–19602 (2013). 24218598 10.1073/pnas.1317010110 PMC3845164

[b37] Walton M. E. & Mars R. B. Probing human and monkey anterior cingulate cortex in variable environments. Cogn. Affect. Behav. Neurosci. 7, 413–422 (2007). 18189014 10.3758/cabn.7.4.413 PMC2519031

[b38] Holroyd C. B. & Coles M. G. H. The neural basis of human error processing: reinforcement learning, dopamine, and the error-related negativity. Psychol. Rev. 109, 679–709 (2002). 12374324 10.1037/0033-295X.109.4.679

[b39] Brown J. W. & Braver T. S. Learned predictions of error likelihood in the anterior cingulate cortex. Science 307, 1118–1121 (2005). 15718473 10.1126/science.1105783

[b40] Amiez C., Joseph J. P. & Procyk E. Anterior cingulate error‐related activity is modulated by predicted reward. Eur. J. Neurosci. 21, 3447–3452 (2005). 16026482 10.1111/j.1460-9568.2005.04170.x PMC1913346

[b41] Matsumoto K., Suzuki W. & Tanaka K. Neuronal correlates of goal-based motor selection in the prefrontal cortex. Science 301, 229–232 (2003). 12855813 10.1126/science.1084204

[b42] Magno E., Foxe J. J., Molholm S., Robertson I. H. & Garavan H. The anterior cingulate and error avoidance. J. Neurosci. 26, 4769–4773 (2006). 16672649 10.1523/JNEUROSCI.0369-06.2006 PMC6674160

[b43] Shima K. & Tanji J. Role for cingulate motor area cells in voluntary movement selection based on reward. Science 282, 1335–1338 (1998). 9812901 10.1126/science.282.5392.1335

[b44] Quilodran R., Rothé M. & Procyk E. Behavioral shifts and action valuation in the anterior cingulate cortex. Neuron 57, 314–325 (2008). 18215627 10.1016/j.neuron.2007.11.031

[b45] Stuss D. T. *et al.* Multiple frontal systems controlling response speed. Neuropsychologia 43, 396–417 (2005). 15707616 10.1016/j.neuropsychologia.2004.06.010

[b46] Cohen R., Kaplan R., Moser D., Jenkins M. & Wilkinson H. Impairments of attention after cingulotomy. Neurology 53, 819–819 (1999). 10489048 10.1212/wnl.53.4.819

[b47] Holroyd C. B. & Yeung N. Motivation of extended behaviors by anterior cingulate cortex. Trends Cogn. Sci. 16, 122–128 (2012). 22226543 10.1016/j.tics.2011.12.008

[b48] Heilbronner S. R., Hayden B. Y. & Platt M. L. Decision salience signals in posterior cingulate cortex. Front. Neurosci. 5, 1–9 (2011). 21541308 10.3389/fnins.2011.00055 PMC3082768

[b49] Jakobs O. *et al.* Across-study and within-subject functional connectivity of a right temporo-parietal junction subregion involved in stimulus–context integration. Neuroimage 60, 2389–2398 (2012). 22387170 10.1016/j.neuroimage.2012.02.037 PMC3321133

[b50] Carter R. M. & Huettel S. A. A nexus model of the temporal–parietal junction. Trends Cogn. Sci. 17, 328–336 (2013). 23790322 10.1016/j.tics.2013.05.007 PMC3750983

[b51] Guyer A. E., Choate V. R., Pine D. S. & Nelson E. E. Neural circuitry underlying affective response to peer feedback in adolescence. Soc. Cogn. Affect. Neurosci. 7, 81–92 (2012). 21828112 10.1093/scan/nsr043 PMC3252630

[b52] Akhrif A., Bajer C., Wohlschläger A. M., Konrad K. & Neufang S. Development-related dynamics in a top-down control network. J. Neurosci. Neuroeng. 2, 255–266 (2013).

[b53] Ossandón T. *et al.* Transient suppression of broadband gamma power in the default-mode network is correlated with task complexity and subject performance. J. Neurosci. 31, 14521–14530 (2011). 21994368 10.1523/JNEUROSCI.2483-11.2011 PMC6703400

[b54] von der Gablentz J., Tempelmann C., Münte T. & Heldmann M. Performance monitoring and behavioral adaptation during task switching: An fMRI study. Neuroscience 285, 227–235 (2015). 25446349 10.1016/j.neuroscience.2014.11.024

[b55] Friston K. J. *et al.* Statistical parametric maps in functional imaging: a general linear approach. Hum. Brain Mapp. 2, 189–210 (1994).

[b56] Brett M., Anton J. L., Valabregue R. & Poline J. B. Region of interest analysis using the MarsBar toolbox for SPM 99. Neuroimage 16, S497 (2002).

[b57] Preuschoff K., Bossaerts P. & Quartz S. R. Neural differentiation of expected reward and risk in human subcortical structures. Neuron 51, 381–390 (2006). 16880132 10.1016/j.neuron.2006.06.024

[b58] McLaren D. G., Ries M. L., Xu G. & Johnson S. C. A generalized form of context-dependent psychophysiological interactions (gPPI): a comparison to standard approaches. Neuroimage 61, 1277–1286 (2012). 22484411 10.1016/j.neuroimage.2012.03.068 PMC3376181

[b59] O’Reilly J. X., Woolrich M. W., Behrens T. E., Smith S. M. & Johansen-Berg H. Tools of the trade: psychophysiological interactions and functional connectivity. Soc. Cogn. Affect. Neurosci. 7, 604–609 (2012). 22569188 10.1093/scan/nss055 PMC3375893

