# Supplementary material for: Neural circuitry involved in quitting after repeated failures: role of the cingulate and temporal parietal junction
Source: Sci Rep. 2016 Apr 21;6:24713. doi: 10.1038/srep24713 (PMC4838821; doi:10.1038/srep24713)

Abbreviated title: Repeated failure on decision making

Neural circuitry involved in quitting after repeated failures: role of the cingulate and temporal parietal junction

Weihua Zhao[1, a], Keith M Kendrick[1, b], Fei Chen[a], Hong Li[c,*] Tingyong Feng[a,d,*]

[a] School of Psychology, Southwest University, Chongqing 400715, China

[b] Key Laboratory for Neuroinformation, Center for Information in Medicine, School of Life Science and Technology, University of Electronic Science and Technology of China, Chengdu 610054, China

[c] School of Psychology and Sociology, Shenzhen University, Shenzhen 518060, China

[d] Key Laboratory of Cognition and Personality, Ministry of Education, Chongqing 400715, China

[1]Weihua Zhao and Keith M Kendrick contributed equally to this work.

[*]Please address correspondence to: Hong Li, School of Psychology and Sociology, Shenzhen University, Nanhai Ave 3688, Shenzhen 518060, China. E-mail: lihongwrm@vip.sina.com; Tingyong Feng, School of Psychology, Southwest University, No. 1 Tian Sheng Road., Beibei, Chongqing 400715, China. Tel: +86 23 68367572. Fax: +86 23 68253629. E-mail: fengty0@swu.edu.cn

Supplementary Information

Results

Relationship between decision confidence and quitting rate

We investigated the relationship between decision confidence and quitting rate using Pearson correlation analysis and found that decision confidence significantly negatively correlated with quitting rate, r=-0.488, p<0.001(see Figure S1). It suggested that quitting rate was influenced by decision confidence.

Using logistic regression to predict the probability of quit the item (quitting rate) after N negative feedbacks. This simplified model used two factors (confidence, negative feedback). The model could be expressed as

$$P(y = quit) = \exp(Am * F + Bn * C) / 1 + \exp(Am * F + Bn * C).$$

Using the following functions: Logit(P)= Am*F+ Bn*C, where F is the number of negative feedback , C is the decision confidence , Am is the regression coefficient of negative feedback and Bn is the regression coefficient of decision confidence. The index of the model were followed: Cox&Snell R^2 =0.302, Nagelkerke R^2=0.472, accuracy of prediction =83.6%. Based on the model, we can draw the ROC curve, which the area under ROC curve was 0.8< S=0.881<0.9 that suggested the model diagnosis was good (S.E.=0.009, p<0.001, 95% confidence interval is 0.875-0.909). Using Hosmer-Lemeshow analysis to test goodness of fit, we found that there was no difference between prediction result and initial data, which indicated that the model was good (χ^2=12.075, df=7, p=0.098>0.05). These results showed that whether subjects decided to continue or quit was influenced by the number of times negative

feedback was received in combination with decision confidence.

Neural correlates of number of negative feedbacks received and quitting rate

Repeated-measures ANOVA was used to test whether the parameter estimates of each target region were significantly different for each of the four frequencies of negative feedback (i.e. whether the pattern of each target region was similar to that observed in the behavioral results). The results showed there were main effects of negative feedback in dACC, PCC and TPJ [$F_{(3,76)}=3.19, p=0.028$; $F_{(3,76)}=4.24$, $p=0.008$; $F_{(3,76)}=5.02$, $p=0.003$), i.e. they showed the same trend as the behavioral results. Post-hoc, Bonferroni-corrected analysis results showed that the parameter estimate for the first negative feedback was significantly different from the second ($p=0.048$), the third ($p=0.01$) and fourth ($p=0.005$) respectively in dACC ($M1=-0.095$, $SD1=0.34$, $M2=0.045$, $SD2=0.43$, $M3=0.21$, $SD3=0.39$, $M4=0.26$, $SD4=0.43$). For PCC ($M1=-0.071$, $SD1=0.25$, $M2=-0.035$, $SD2=0.25$, $M3=-0.15$, $SD3=0.12$, $M4=-0.24$, $SD4=0.16$), there were no significant differences between the first, second and third feedbacks ($p>0.05$) but there was for the fourth one ($p=0.037$). For the TPJ ($M1=-0.063$, $SD1=0.26$, $M2=-0.21$, $SD2=0.28$, $M3=-0.32$, $SD3=0.31$, $M4=-0.39$, $SD4=0.29$) there were no differences between the first two times of negative feedback ($p=0.119$) but there were between the first and the third and fourth times ($p=0.007$, $p=0.005$, see Figure S2 a, c, d).

Exploring the relationship between parameter estimates for the three ROIs (dACC, PCC and TPJ) using the linear regression results suggested that dACC activation was associated with predicting subsequent choices after different numbers of negative

feedbacks. During the first negative feedback, when the majority of subjects chose to continue, the slope was not significant (*slope*=1.19, p=0.17). However, dACC activation could predict quitting rates after the second (y=10.86x+3.49, *slope* =10.86, p=0.002) and the third (y=22.71x+21.95, *slope*=22.71, p=0.05) negative feedbacks but not after the fourth when the majority of subjects chose to give up (*slope*=0.44, p=0.93, see Figure S2b).

Legends

Figure S1. Decision confidence results. The relationship between the decision confidence and quitting rate. Decision confidence negatively correlated with the number of negative feedbacks. Error bars indicate standard errors of the mean.

Figure S2. ROI results. a,c,d, Brain activations were extracted from an 8 mm diameter spherical dACC, PCC, TPJ ROIs. Histograms show mean±sem dACC (a), PCC(c), and TPJ(d) parameter estimates across the four frequencies of negative feedback. b, Scatterplot of a regression analysis between neural parameter estimates of dACC and quitting rate across the four frequencies of negative feedback. * $p<0.05$, ** $p<0.01$

Figure S1

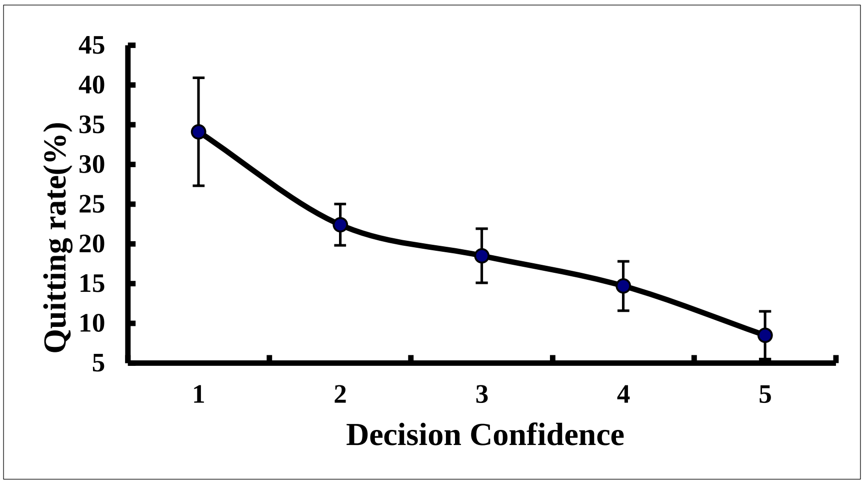

Figure S2

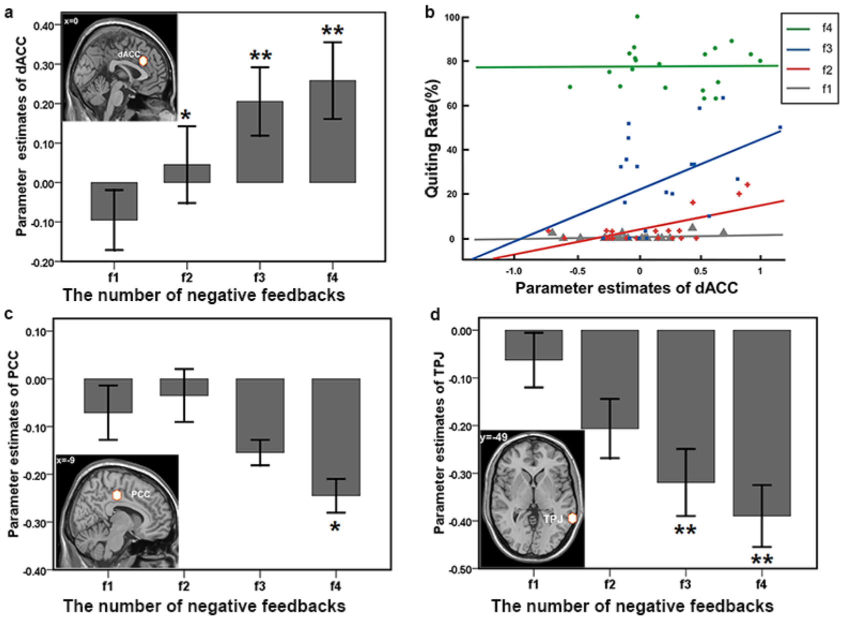

Supplement: Supplementary Information [file srep24713-s1.pdf]
